# Protein stabilization with retained function of monellin using a split GFP system

**DOI:** 10.1038/s41598-018-31177-z

**Published:** 2018-08-24

**Authors:** Tanja Weiffert, Sara Linse

**Affiliations:** Department of Biochemistry and Structural Biology, Chemical Centre, Lund University, SE221 00 Lund, Sweden

## Abstract

Sweet proteins are an unexploited resource in the search for non-carbohydrate sweeteners mainly due to their low stability towards heating. Variants of the sweet protein monellin, with increased stability, were derived by an *in vivo* screening method based on the thermodynamic linkage between fragment complementation and protein stability. This approach depends on the correlation between mutational effects on the affinity between protein fragments and the stability of the intact protein. By linking the two fragments of monellin to the split GFP (green fluorescent protein) system, reconstitution of GFP was promoted and moderately fluorescent colonies were obtained. Two separate random libraries were produced for the monellin chains and the mutant clones were ranked based on fluorescence intensity. Mutants with increased affinity between the fragments, and subsequently increased stability, caused increased fluorescence intensity of split GFP. Single chain monellin variants of the top-ranked mutants for each chain, S76Y in the A-chain and W3C + R39G in the B-chain and all combinations thereof, were expressed and the increase in stability was verified by temperature denaturation studies using circular dichroism spectroscopy. Functionality studies showed that mutant S76Y has retained sweetness and has potential use within the food industry.

## Introduction

Stability toward denaturation is of utmost importance for proteins to be used in a technical application, within the pharmaceutical industry or food industry. Proteins should endure handling, heating and storage. Evolution has made proteins marginally stable towards denaturation limiting the usefulness of proteins in industrial processes. Stability of proteins is dependent on various non-covalent interactions such as hydrogen bonds, van der Waals interactions, hydrophobic interactions and electrostatic interactions, within the protein and between protein and solvent molecules. The net sum of these interactions in the folded state minus the net sum in the unfolded state gives the stability of a protein. Proteins can thus be stabilized either through structural changes or by changing the composition or properties of the solvent surrounding them^1^. Several approaches to prevent protein degradation through formulations with sugars, polyols, polymers, surfactants and salts (reviewed in ref.^2^) have been developed. Structural modifications such as glycosylation and polyethylene glycolylation (PEGylation) can increase the protein stability and numerous procedures to obtain proteins with increased stability by altering the amino acid sequence have been explored, e.g. directed evolution (reviewed in ref.^3^), rational design (reviewed in ref.^4^), phage display (review in ref.^5^) and computational approaches^6^.

Here we use an *in vivo* screening method based on split green fluorescent protein (GFP) and the thermodynamic linkage between fragment complementation and protein folding, first shown by Lindman *et al*.^7^, to develop mutants of the sweet tasting protein monellin (MN) with high stability. The method relies on the correlation between mutational effects on the affinity between fragments of a protein and effects on the stability of the intact protein. In this approach, the protein of interest is split into two fragments that are linked to split GFP. In the case of monellin, that naturally consists of two non-covalently bound chains, the two chains are attached to split GFP. Protein variants with increased affinity between the fragments (and subsequently increased stability) cause increased fluorescence intensity of split GFP by favoring reconstitution/folding of GFP and thereby chromophore maturation. This approach enables selection of mutations in the protein of interest that favor the associated state more than the dissociated state as well as mutations that disfavor the dissociated state more than the associated state. Such *in vivo* selection of stabilized protein variants based on fluorescence intensity allows stabilization of proteins without additives and minimizes the risk of selection due to surface activity, which is a potential problem in phage display. The facile and affordable split GFP based method can be applied to both unbiased libraries produced by error-prone polymerase chain reaction (PCR) and to rationally designed libraries. Lindman *et al*.^7^ used the split GFP system to screen for B1 domain of protein G (PGB1) mutants with increased stability from a designed library as proof of concept of the approach. Here we report the first unbiased fragment libraries screened with this method. Furthermore, we couple the screen for stabilized variants to a functional readout and demonstrate the importance of such validation of functionality of the stabilized protein.

The two fragments of split GFP do not reconstitute spontaneously and consequently do not emit any fluorescence when co-expressed. By fusing the fragments of GFP to other molecules that interact, reconstitution can be promoted and fluorescence obtained. The fused molecules facilitate complex formation of the fluorescent fragments by bringing them in proximity to each other. Regan and co-workers showed that an affinity above ca. 10^3^ M^−1^ between the fused molecules is sufficient for detectable green fluorescence to emerge^8^, whereas high affinity above 10^9^ M^−1^ promotes very bright fluorescence^9^. The main fields of use of split GFP and other bimolecular fluorescence complementation (BiFC) assays are to study protein interactions and to visualize the subcellular localization of molecular interactions^10^. Fragments of several different proteins have been used to detect protein interactions, e.g. ubiquitin, β-galactosidase, luciferases and different versions of GFP^11–14^, though fluorescent proteins remain the most common approach. GFP emits bright green fluorescence when folded correctly and it obtains its native fold slowly through several intermediates^15^. The protein has been mutated (F64L, S65C, Q80R, Y151L, I167T, and K238N) to get one excitation peak at 475 nm and emission at 505 nm^16^. The chromophore consists of residue Cys65-Tyr66-Gly67 and is in the interior of a β-barrel structure. Split GFP linked to monellin fragments results in moderate fluorescence which enables detection of monellin variants with increased stability.

Sweet proteins acquire attention as there is a zeal for non-carbohydrate sweeteners from food industry. Sweeteners within food industry must maintain their sweetness at high temperature, high pressure and at varying pH. Monellin has an intense and persistent sweetness and is approximately 100 000 times sweeter than sucrose on a molar basis^17^. The protein belongs to a family of seven structurally heterogeneous, sweet and sweet taste-modifying proteins: monellin^18^, brazzein^19^, thaumatin^20^, mabinlin^21^, lysozyme^22^, neoculin^23^ and miraculin^24^. Monellin was originally isolated from the tropical serendipity berry (*Dioscoreophyllum cumminsii*)^18^ and the sweetness of monellin can be sensed by old world monkeys and humans only^25^. The protein interacts with the human sweet taste receptor T1R2-T1R3 (a heterodimeric G-protein coupled receptor (GPCR)) on the tongue^26–28^. The exact mode of interaction between sweet proteins and the receptor is unknown but the “wedge model” has been proposed to explain how sweet proteins stabilizes the active form of the receptor by binding to an external cavity spanning the T1R2-T1R3 dimer^29,30^. The sweetness of proteins depends on their net charge, surface charge distribution and the three-dimensional structure of the protein and thereby its surface^31–33^. Monellin is a heterodimer of two subdomain fragments interacting tightly in a single domain with a β-grasp fold, which consists of an α-helix packed perpendicular to a five-stranded anti-parallel β-sheet^17,34^. A single-chain derivative of monellin (scMN) is used in this study. The sweetness is retained in scMN and it has increased stability compared to wild type monellin heterodimer^35–37^. However, after heating or boiling, scMN may not refold reversibly depending on the time spent at high temperature, and on the protein concentration, due to sample aggregation and precipitation^38^. Here we therefore utilize the split GFP assay with the monellin subdomain fragments to obtain an even more stable variant of scMN, with retained sweetness, that can meet the stability requirements from the food industry.

## Results

### Nomenclature

**Figure 1:**
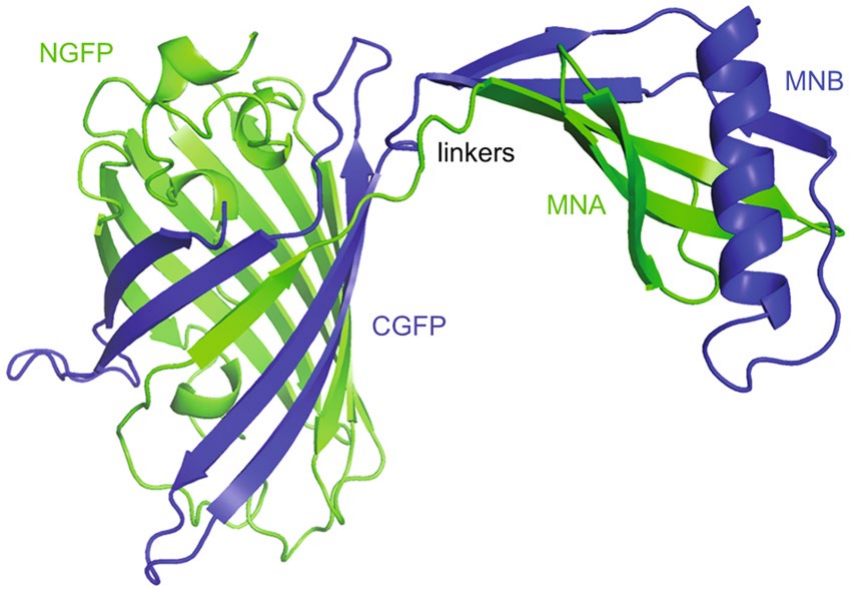
A cartoon model of split GFP fused to monellin. MNA was fused to NGFP (green) and MNB was fused to CGFP (blue). The model is produced from pdb files 1EMA^54^ and 2O9U^55^ using Pymol^56^.

Wild type monellin consists of two non-covalently attached chains, MNA and MNB^17^. The split GFP system used here relies on non-covalent association of the NGFP-MNA and MNB-CGFP gene products. NGFP-MNA denotes a fusion construct where the C-terminus of the N-terminal GFP fragment (residues 1 to 157) has been linked through an eight-residue linker to the N-terminus of MNA. MNB-CGFP is a fusion construct where the C-terminus of MNB has been linked to the C-terminal fragment of GFP (residues 158 to 238) through a seven-residue linker (Fig. 1). Parent MN (pMN) denotes a construct where cysteine 41 in the B-chain has been replaced by serine to avoid disulfide bridges. ScMN denotes a single-chain construct of monellin where the C-terminus of the B-chain (MNB) has been linked to the N-terminus of the A-chain (MNA) by a Gly-Phe linker.

### Expression of NGFP-MNA and MNB-CGFP constructs

**Figure 2:**
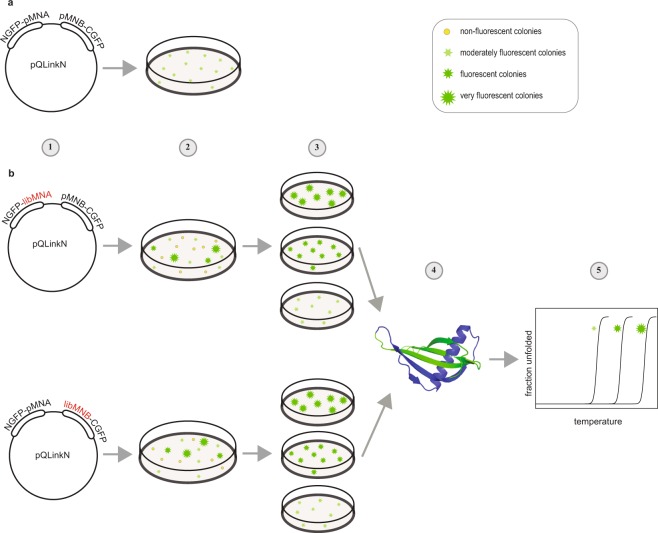
Workflow of *in vivo* selection of monellin mutants with increased stability. (**a**) Expression of parent MN on inducing plates. (**b**) Production of stabilized MN mutants. Step 1: Random library production of the A (libMNA) and B (libMNB) chain of MN in the pQLinkN plasmid by error-prone PCR. Step 2: Selection of mutant clones with increased fluorescence compared to split GFP-pMN. Step 3: Ranking of mutant clones on individual inducing plates with increased fluorescence. Step 4: Production of single chain parent monellin and mutants. Step 5: Verification of increased stability of single chain mutants by temperature denaturation.

**Figure 3:**
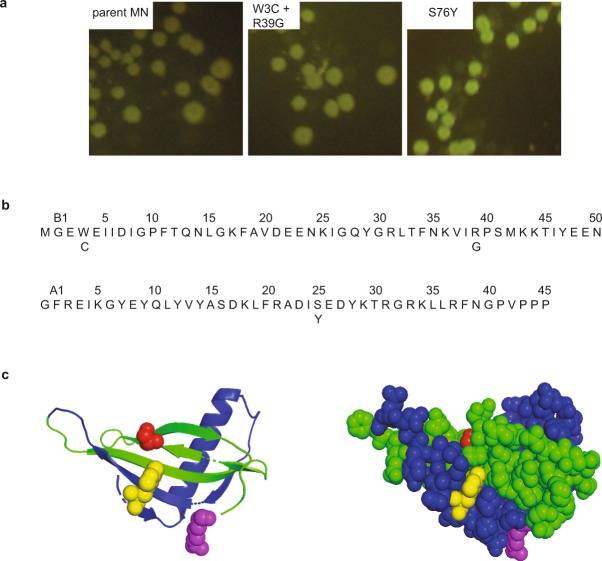
Result of fluorescence screening of fragment complementation. (**a**) Comparison of fluorescence intensity of parent MN, W3C + R39G and S76Y on inducing plates. (**b**) Amino acids sequence of parent MN with top ranked mutations shown below the sequence. (**c**) Structure of parent MN, ribbon model (left) and space-filling model (right) with the sites for top ranked mutations, W3 (yellow), R39 (purple) and S76 (red), shown as space filling models. The structures were produced from pdb file 2O9U^55^ using Pymol^56^.

The genes for NGFP-MNA and MNB-CGFP were fused to the same plasmid by ligation independent cloning (LIC) to ensure similar expression levels of the two constructs^39^. Transformation of this plasmid into *Escherichia coli* (*E*. *coli*) and expression of the parent NGFP-MNA and MNB-CGFP, under inducing conditions, resulted in green fluorescent colonies after approximately 2–4 days at room temperature (Figs 2 and 3a). Culturing on non-inducing plates showed no green fluorescence; neither did a negative control on inducing plates expressing NGFP and CGFP without MNA and MNB.

### Ranking of fluorescent monellin mutants with increased stability

We set out to search for split GFP library colonies with increased green fluorescence compared to colonies containing the combined parent fragments, NGFP-MNA and MNB-CGFP. This was motivated by the correlation between the stability of the singe-chain protein and the affinity between protein fragments; the MN fragment association serving as a driver of GFP fragment association, folding and maturation of its chromophore. Two separate random libraries were produced for the A and B chain of monellin, respectively (Fig. 2). The MNA library was co-expressed with parent MNB and the MNB library was co-expressed with parent MNA. The Mutazyme® II DNA polymerase was chosen to obtain similar mutation frequency for all nucleotides. The desired mutation level of 1–2 mutations per monellin chain was verified by DNA sequencing, though some triple mutations were also found. The libraries where spread on inducing plates and plasmids were purified from colonies identified with increased fluorescence intensity compared to the split GFP reconstituted with parent monellin chains. Selected mutant clones were expressed on separate inducing plates and ranked based on fluorescence intensity and compared to parent MN. The ranking was performed by six volunteers and the top 30 ranked mutants were sequenced. The two selected top ranked mutations were S25Y in the A chain of monellin (which corresponds to S76Y in single chain monellin) and W3C + R39G in the B chain of monellin (which corresponds to W3C and R39G in single chain monellin) (Fig. 3). The brightest fluorescence was emitted by split GFP fused to the S76Y mutant.

The S76Y mutation was also found in a double mutant (MNA L11R + MNA S25Y) as well as in a triple mutant (MNA S25Y + MNA P43S + MNA P45S). Arginine at position 39 in the B chain of monellin was found to be mutated to five different amino acids (G, C, S, H, L) within the 30 top ranked colonies. The W3C mutation was not found as a single mutation but the tryptophan was replaced by a glycine in one of the top ranked mutants. Other mutants from the top ranked colonies with increased fluorescence were F38S, D27N, F1S, V42I, N39Y, P43L, K18I + A22V, G32D, F1L, F1T + G32D, Y12stop for MNA and E2D, N14K, G1S, E22D, F18Y, R39H + K43I, E48V for MNB.

In an attempt to increase the stability of MN even further, a random library of MNB was expressed together with the MNA S25Y mutant but no mutants with increased fluorescence intensity compared to MNA S25Y mutant with parent MNB were found.

### Protein expression and purification of single chain monellin mutants

**Figure 4:**
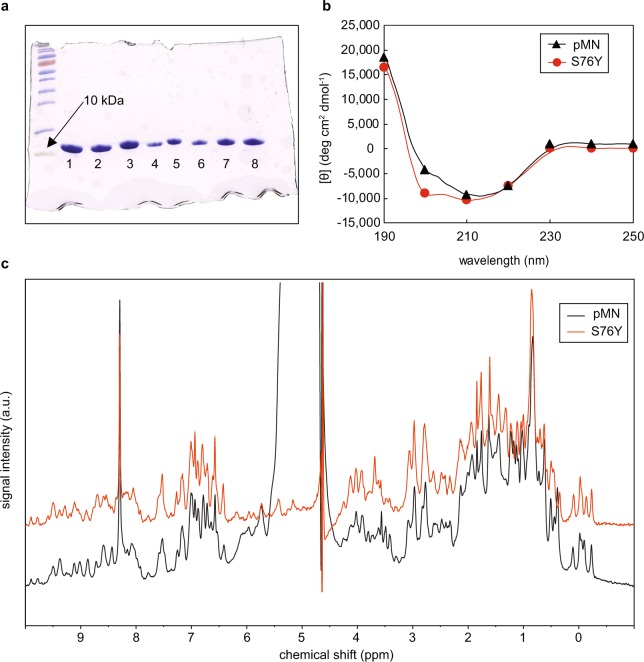
Protein homogeneity and structural analysis of single chain parent monellin and mutants. (**a**) SDS-PAGE (15%) of PageRuler™ Prestained protein ladder, 10–180 kDa, to the left, purified parent scMN (lane 1), S76Y (lane 2), W3C (lane 3), R39G (lane 4), W3C + S76Y (lane 5), R39G + S76Y (lane 6), W3C + R39G (lane 7) and BW3C + R39G + S76Y (lane 8). (**b**) Far UV spectra of parent scMN (black solid line and triangles) and S76Y (red line and circles) in 5 mM sodium phosphate buffer, pH 5.5, 20 °C. Data is shown as lines with symbols at every tenth data point. (**c**) 1D NMR spectra of parent MN (black) and S76Y (red) in H_2_O with 10% D_2_O, pH 6.25.

Single chain monellin variants (devoid of GFP) were produced to enable verification of increased stability of the mutants by thermal denaturation experiments. Parent scMN, single mutants (W3C, R39G and S76Y), double mutants (W3C + S76Y, W3C + R39G and R39G + S76Y) and the triple mutant (W3C + R39G + S76Y) were expressed in *E*. *coli* and purified using weak cation exchange chromatography and gel filtration. Dithiothreitol (DTT) was added to variants containing the W3C mutation to retain monomeric samples. Sodium dodecyl sulfate polyacrylamide gel electrophoresis (SDS-PAGE) analysis revealed that highly pure protein was obtained for all mutants (Fig. 4a).

### Mass spectrometry (MS)

Length and identity of the proteins were verified by mass spectrometry. Parent scMN and mutants were cleaved by trypsin and investigated by MS and MSMS. For parent monellin and mutants W3C, S76Y, W3C + S76Y, W3C + R39G and W3C + R39G + S76Y, both full length protein and protein where the N-terminal methionine was cleaved off were found. For example, both peptide MGEWEIIDIGPFTQNLGK and GEWEIIDIGPFTQNLGK were found from parent scMN.

### Secondary structure by farUV circular dichroism (CD) spectroscopy

Secondary structure of parent scMN and S76Y was evaluated by CD spectroscopy in the far-UV region (Fig. 4b). The spectra suggest that both proteins are folded and that the global fold is similar. The minima observed at approximately 200–210 nm in spectrum for the S76Y mutant can most likely be ascribed to the addition of an aromatic residue^40^. The changes in intensity are likely due to concentration differences.

### Nuclear magnetic resonance (NMR) spectroscopy

NMR spectra revealed highly pure protein with well-folded structures for parent scMN and S76Y, (Fig. 4c). Spectra for both proteins have similar line width and similar distribution of peaks. There are only minor differences in chemical shifts. This indicates that the structures of parent scMN and S76Y are very similar. The peak observed at 8.5 ppm originates from the NMR tube.

### Thermal stability of single chain monellin mutants

**Figure 5:**
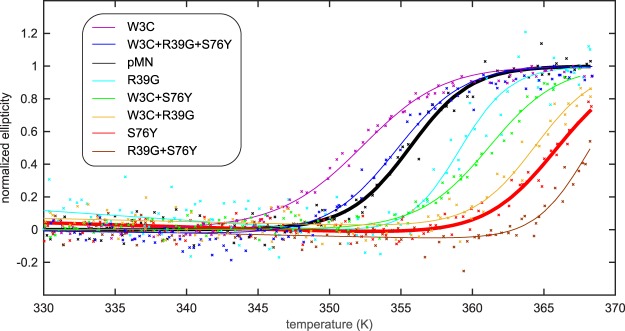
Thermal denaturation of single chain monellin. Normalized thermal denaturation monitored by CD spectroscopy at 215 nm of W3C (purple), W3C + R39G + S76Y (blue), parent scMN (black), R39G (light blue), W3C + S76Y (green), W3C + R39G (orange), S76Y (red), R39G + S76Y (brown) in 5 mM sodium phosphate buffer, pH 5.5. The fits to each data set are shown as solid lines.

The stabilities of top ranked mutants and parent scMN were examined in 5 mM sodium phosphate buffer, pH 5.5 (with addition of 1 mM DTT for cysteine-containing mutants) by thermal denaturation monitored by the CD signal at 215 nm (Fig. 5). For all protein variants, a loss in signal intensity was recorded as the temperature increased. The denaturation data appear to follow a sigmoidal curve, indicating cooperative denaturation. Previous studies have shown that temperature denaturation of parent scMN is reversible when the protein is briefly heated to 95 °C, while prolonged heating causes aggregation and precipitation^38^. Since monellin has been shown to be aggregation prone, the protein concentration was kept low (6 µM) during temperature denaturation experiments. Reverse thermal scans indicate that the denaturation process is reversible for parent scMN, S76Y, W3C and W3C + S76Y. The sigmoidal part of the curve is reversible for all samples, though mutants R39G, R39G + S76Y, W3C + R39G and the triple mutant W3C + R39G + S76Y experience a small gain of signal during the reverse scan (Fig. S1). The most stable monellin variants did not reach the final plateau of the sigmoidal curve, indicating that the plateau lies beyond 95 °C. The midpoint of thermal denaturation (T_m_) (which is the primary variable of interest), enthalpies of unfolding (ΔH°_Tm_) and the free energies of unfolding (ΔG°) (Table 1), were acquired by fitting equations (1) and (3) to the data.

**Table 1:** Thermodynamic parameters of parent MN and mutants.

Protein	T_m_ (°C)	ΔH°_Tm_ (kJ/mol)	ΔG° (25 °C) (kJ/mol)	ΔT_m_ (°C)
parent MN	83 ± 1	460 ± 40	46 ± 6	—
W3C	79 ± 1	390 ± 30	34 ± 5	−4
R39G	85 ± 2	490 ± 90	51 ± 16	2
S76Y	93 ± 2	560 ± 90	64 ± 17	10
W3C + R39G	91 ± 0.2	550 ± 50	63 ± 9	8
W3C + S76Y	89 ± 1	480 ± 60	49 ± 10	6
R39G + S76Y	96 ± 1	910 ± 380	132 ± 74	13
W3C + R39G + S76Y	83 ± 2	480 ± 100	49 ± 16	0

The errors are the standard deviations obtained from three experiments.

The mid-point of thermal denaturation of parent scMN is 83 °C, which correlates well with previously published data^38^. Top-ranked mutations, S76Y and W3C + R39G, obtained from the ranking of the fluorescent GFP-MN variants, contributed to increased stability compared to parent scMN. The stability order of the mutants is the same as the order of the green fluorescence intensity, the top ranked S76Y being the most stable. The single mutation R39G may slightly increase the stability; however, the single W3C mutant have decreased stability and the triple mutant (W3C + R39G + S76Y) have unchanged stability. The most stable variant is the double mutant R39G + S76Y, where the midpoint of thermal denaturation has increased with 13 °C compared to parent scMN. The double mutant W3C + S76Y also experience higher stability than parent scMN. Due to the homogeneity caused by the presence/lack of the N-terminal Met in single-chain monellin the reported values of ΔH°_Tm_, T_m_ and ΔG° are averages for protein with different termini.

### Sweetness detection

The sweetness of parent scMN, R39G, S76Y and R39G + S76Y were evaluated by six volunteers. Monellin concentrations ranging from 19 to 5607 nM were tested. Sweetness of sucrose was detected by all testers. One tester judged MQ water (negative control) as sweet and was therefore discarded from the study. Parent scMN and S76Y were recorded as sweet by all testers (Table S1). The average sweetness threshold for parent scMN and S76Y were 317 nM (219–492 nM) and 263 nM (219–328 nM), respectively, which is comparable to previously published data for wild type single chain monellin, natural monellin and wild type synthetic monellin^31,37,41,42^. In accordance to earlier studies, monellin was approximately 100 000 times sweeter than sucrose on a molar basis. Two test people did not detect any sweetness for R39G + S76Y and three judged the samples with the second highest or the highest concentration as sweet. Sweetness of R39G was sensed at high concentrations (1108–5607 nM) for all testers. The sweetness of R39G was confirmed by one volunteer at a concentration of 12 µM.

## Discussion

Proteins are marginally stable, the free energy of the folded state of a protein lying only 20–60 kJ mol^−1^ below that of the unfolded state. This may reduce the usefulness of proteins for industrial purposes, e.g. as pharmaceuticals or food. Hence it is of great interest to stabilize proteins. Since many proteins in nature are optimised for function rather than stability, there is often room for increased stability; however, it is critical that the stabilizing mutations do not impede the function of the protein. Protein stability is defined as the free energy difference between the folded and unfolded state. Mutations increasing the energy of the unfolded state more than the folded state, as well as mutations decreasing the free energy of the folded state more than the unfolded state, are thus stabilizing. To design more stable mutants remains hard despite that key factors influencing protein stability, such as packing of the hydrophobic core, hydrogen bond formation, electrostatic interactions, conformational entropy and bond strain are rather well understood^43–45^. Here we utilize the thermodynamic linkage between fragment complementation and protein stability to find more stable variants of monellin. Mutations affecting the affinity between fragments also affect the stability of the protein. The correlation between fluorescence intensity of split GFP and fragment affinity of the attached protein fragments thus enables *in vivo* selection of proteins with enhanced stability. Improved stability of monellin may allow heating to higher temperatures which aids industrial usage of the protein. Monellin was chosen for this study since it naturally consists of two chains that reconstitute the protein spontaneously. The complementation rate is slow with an association rate constant, k_on_ of 8.8 × 10^3^ M^−1^ s^−1^, and a dissociation rate constant, k_off_ of 3.1 × 10^−4^ s^−1^, resulting in an equilibrium dissociation constant (K_D_) of 35 nM^46^. Fragment complementation of NGFP-MNA and MNB-CGFP results in moderately fluorescent colonies which allows for detection of colonies with increased fluorescence intensities. Detection of colonies with increased fluorescence intensity was performed by human eye. The human eye can capture differences in fluorescence intensity between colonies better than a fluorescence plate reader. However, this is a time-consuming step which limits the number of screened colonies. To further improve the efficiency of this method, fluorescence-activated cell sorting (FACS) might be used as a ranking method.

According to the *in vivo* fluorescence ranking the most stabilizing mutation was S76Y, which was confirmed by temperature denaturation studies. S76Y retained its functionality and was considered as sweet as parent monellin. The increased stability is likely due to the additional van der Waals interactions formed by the tyrosine and its surrounding amino acids. Tyrosine residues are more often buried within proteins than serine residues and generally have more favourable van der Waals interactions of the aromatic ring compared to serine, whereas both can participate in hydrogen bonds via the -OH group^47^. S76 is situated in the middle of the β4 strand and tyrosine has higher β-sheet forming propensity than serine which may also increase the stability of the mutant^48^. The serine residue is placed in a pocket on the protein surface and tyrosine might fill the pocket better. Tyrosine in this position may thus stabilise the folded state relative to the unfolded state in which residue 76 is likely fully exposed to solvent. The double mutations W3C + R39G were the most stabilizing mutations found in the B-chain of monellin according to the *in vivo* fluorescence ranking. Increased stability of the mutant was verified by temperature denaturation where the W3C + R39G mutant had an increased T_m_ by approximately 8 °C compared to parent scMN. The mutations were also examined separately and the single mutations did not improve stability to any great extent. The single W3C mutation reduced the stability and the R39G mutation had minor, if any effect on the stability. The positively charged arginine 39 is exposed on the surface of the protein and mutation to neutral glycine may be stabilizing by decreasing local electrostatic repulsion. In five out of the top thirty variants with increased fluorescence intensities R39 was mutated, implying that R39 has negative effect on the stability. Introduction of W3C mutation removes a large hydrophobic residue from the surface of the protein which may influence the stability. Addition of reducing agents during temperature denaturation studies minimize the risk of dimerization (of mutants containing W3C) affecting the stability. The likelihood of dimerization in the *in vivo* assay is low due to the linkage of the monellin fragments to the bulky GFP molecule, though it cannot be ruled out.

**Figure 6:**
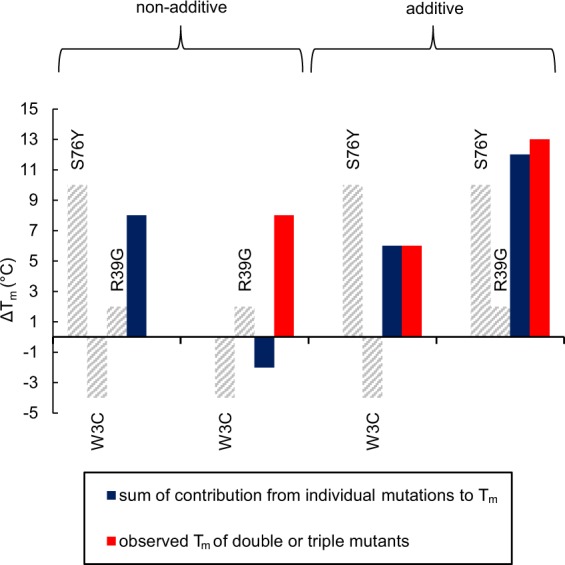
Analysis of additivity of mutational effects. Non-additive and additive effects on T_m_ of the selected mutations. Contribution of individual mutations to T_m_ are shown in grey, sum of contribution from individual mutations to T_m_ are shown in blue and observed T_m_ of double or triple mutants are shown in red. The contribution of the individual T_m_ values and the observed T_m_ of double or triple mutants are adapted from Table 1.

Mutational effects on protein stability can be additive or non-additive. The stability of monellin with all combinations of mutations were investigated by temperature denaturation which showed that the W3C + R39G and W3C + R39G + S76Y mutations have non-additive effects on the stability while the effects of W3C + S76Y and R39G + S76Y were additive (Fig. 6). S76Y is situated on the opposite side of the protein compared to W3C and R39G, (Fig. 3), resulting in the additive effects of S76Y together with either W3C or R39G. Non-additivity may occur when the mutated residues are in contact with each other. The residues can be in direct contact or through electrostatic interactions. Structural rearrangements propagated by intervening residues can also cause non-additivity effects. By using unbiased libraries we were able to find mutations with non-additive effects that would not be present in a rationally designed library.

As expected, the mutation of arginine 39 resulted in a dramatic decrease of sweetness. The importance of a charged surface to retain sweetness of monellin has been shown repeatedly^31^. Sweet assessment studies have shown that the substitution of arginine 39 with glutamic acid or aspartic acid produced mutants with detection thresholds at 13 000 nM and 89 000 nM, respectively^49^. According to the “wedge model” R39 together with D7, R88 and R65 are crucial to retain sweetness^50^. Our findings correlates well with the wedge model in which arginine 39 interacts with the sweet taste receptor while serine 76 is situated far away from the interaction site^32^. A meta-analysis of the sweetness of published mutants of monellin by Xue *et al*.^31^ showed a correlation between reduced net positive charge and reduced sweetness. Variants with added negative charge always showed reduced sweetness (R39D, R39E, K43E, R72E, R88E, F34D, M42E, Y63E, Y65E). Replacement of a negative residue with a neutral residue showed no change in sweetness detection levels (E2N, MNA D16N (in two-chain monellin), MNA D22N, MNA E25Q, MNA D26N, E59C) except for D7N that experience reduced sweetness. No variation of sweetness was detected when a positively charged residue was replaced by another positively charged residue (R39K) neither did the C41S mutation effect the sweetness of monellin. Other mutations that decrease the sweetness of monellin were D7E (charge-neutral), M42R and D68R (addition of positive charge). Two mutants with increased positive charged (Y63R, Y65R) showed no variation in sweetness. Most mutational studies have aimed to shed light on the interaction of monellin and the sweet taste receptor though some also intend to increase the stability of the protein. Mutation E23Q increased the sweet taste of monellin^50^. A variant of the E23Q mutant with increased stability was constructed by Leone *et al*.^50^, by rational design, which contained Q28K, C41S and Y65R mutations. Removal of the buried ionizable glutamic acid 23 is expected to improve protein stability and a study showed that by mutating it into alanine the stability became pH independent between pH 4 and 8. An additional mutation, C41A, resulted in the stability being pH independent from pH 4 to 10^51^. Arginine 39 has been extensively mutated and although focus has been on its effect on sweet taste, one study showed that the double mutant D7E + R39K had decreased stability towards temperature denaturation^49^.

 The present study corroborates the usefulness of an *in vivo* protein stabilization method based on fragment complementation and a split GFP system for protein stabilization and adds a functional assay to the work-flow. Several novel mutations with increased stability were found, one of which have retained sweetness. However, the top hit for the B-chain of monellin, W3C + R39G, is not suitable as a sweetener. The removal of the positively charged arginine results in decreased sweetness and for industrial purposes cysteine residues are not applicable due to the need of reducing agents to maintain monomeric protein. The functional mutant S76Y, which was the top hit among the A-chain substitutions of monellin in the *in vivo* screen, has similar structure to parent scMN and has potential use as a low-calorie sweetener. Improving the stability of monellin increases its utility in industrial processes where heating is a key procedure. Stabilized variants of monellin can hopefully meet the demand of a non-carbohydrate sweetener for the food industry in the future.

## Materials and Methods

### Construction of a tandem NGFP-MNA-CGFP-MNB pQLinkN plasmid

The original pQLinkN plasmid was a kind gift from Konrad Buessow (Addgene plasmid # 13670)^52^. The cloning procedure is described in Supplementary Information.

### Construction of a library of mutant MNA and MNB

Two libraries of mutant MNA and MNB, respectively, were produced by error prone PCR using GeneMorph II Random mutagenesis kit (Agilent Technologies). By varying the template amount in the PCR reaction, the mutation frequency can be controlled. Mutation levels of 1–2 mutations/gene were desired and the primers used were epNGFPpQLinkNfor, epNGFPpQLinkNrev, epCGFPpQLinkNfor and epCGFPpQLinkNrev, with sequences as listed in Supplementary Information. To remove parent MNA from the NGFP-MNA-CGFP-MNB pQLinkN plasmid and to enable ligation of mutated MNA, the plasmid and the PCR product were separately digested at 37 °C with KpnI (Thermo Scientific) over night, purified with GeneJET PCR purification kit (Thermo Scientific) and digested with SacI (Thermo Scientific) at 37 °C for 1 h. For the last 15 min of the digestion, FastAP Thermosensitive alkaline phosphatase (Thermo Scientific) was added. To enable insertion of mutant MNB, the PCR product and the plasmid were separately digested at 37 °C with NdeI and PstI overnight and FastAP Thermosensitive alkaline phosphatase was added for the last 15 min. The digestion products were purified using GeneJET PCR purification kit (Thermo Scientific). The mutated MNA och MNB fragments were ligated into the appropriately digested plasmid by using T4 DNA ligase (NEB) for 16 h at 16 °C.

### Ranking of fluorescent split GFP-monellin mutants

Plasmids containing parent MNB and mutated MNA, or parent MNA and mutated MNB, were transformed into *E*. *coli* ER2566 and plated on inducing plates (luria broth (LB)/agar plates containing 100 µg/mL ampicillin and 10 µM of Isopropyl β-D-1 galactopyranoside (IPTG)). A plasmid containing parent NGFP-MNA and MNB-CGFP was transformed in parallel for comparison of fluorescence intensity. The plates were kept at 37 °C during 16 h followed by 2–4 days in room temperature. Fluorescent colonies were monitored using a transilluminator with light emission between 420 and 500 nm (Dark Reader DR45M nonUV blue light transilluminator; Clare Chemical Research, Inc.). Plates were imaged using a digital CCD camera (Sony Cybershot DSC-W5). The fluorescence intensity was judged by human eye. Colonies with higher fluorescence intensity than parent MN were picked and cultivated overnight in 2 mL LB with 100 µg/mL ampicillin, followed by plasmid preparation using Illustra plasmid preparation kit (GE Healthcare). Forty colonies with increased fluorescence compared to the parent were selected. Finally, the plasmids prepared from the selected colonies were retransformed into *E*. *coli* ER2566 and plated on individual inducing plates and the colonies were ranked independently by 6 human volunteers based on fluorescence intensity. The 30 top ranked mutants were sequenced using BigDye Terminator v1.1 Cycle sequencing Kit (Applied Biosystems). The primer 5′-C AAC ATT CTG GGA CAC AAA TTG G-3′ was used to sequence MNA and 5′-TGC TAG TTG AAC GCT TCC ATC TTC-3′ was used to sequence MNB (purchased by BM Unit). The top ranked MNA mutant was co-expressed with the MNB library to find mutants with even higher stability.

### Expression and purification of single chain monellin mutants

Pet3a plasmids containing genes for single chain monellin with single (R39G, W3C, S76Y), double (R39G + S76Y, W3C + S76Y, W3C + R39G) or triple (W3C + R39G + S76Y) mutations were purchased from Genscript (Piscataway, New Jersey). Plasmids containing parent scMN and mutants were transformed into Ca^2+^ competent BL21(DE3) Ply Star *E*. *coli* cells and cultivated on LB agar plates containing 50 µg/ml ampicillin and 30 µg/ml chloramphenicol. Single colonies of each mutant were used to inoculated 5 ml LB containing 50 µg/ml ampicillin and 30 µg/ml chloramphenicol at 37 °C overnight. The overnight cultures were used to inoculate 500 ml LB containing 50 µg/ml ampicillin and 30 µg/ml chloramphenicol. Protein expression was induced by addition of 0.4 mM IPTG at OD600 = 0.6. The cultures were harvested after 3–4 hours by centrifugation and the pellet was resuspended in 10 ml of water or 2-(N-morpholino)ethanesulfonic acid (MES) buffer pH 6 (with addition of 1 mM DTT for plasmids containing the W3C mutation) and frozen at −20 °C overnight. The resuspension was thawed on ice, diluted with approximately 30 ml of MES buffer pH 6 (with addition of 1 mM DTT for plasmids containing the W3C mutation) and sonicated for 5 min, 30 s on/30 s off, followed by centrifugation at 16 000 rpm for 20 min. The supernatants were pooled, DNAse was added and the supernatant was left on ice for 15 min, then centrifuged at 16 000 rpm for 20 min. The supernatant was concentrated on a Vivaspin 20 filter, MWCO 3000 (Sartorius). The protein was added to a 5 ml HiTrap CM FF column (GE Healthcare) and washed with 10 mM MES buffer pH 6. The sample was eluted using a 0–0.25 M linear NaCl gradient. Fractions containing monellin mutants were pooled, concentrated and separated on a Superdex 75 (10/300) gel filtration column (GE Healthcare). The running buffer was 5 mM sodium phosphate, pH 5.5, with addition of 1 mM DTT for proteins containing the W3C mutation. The residual salt was removed by gel filtration using a Nap10 column (GE Healthcare) in 5 mM sodium phosphate buffer, pH 5.5, with addition of 1 mM DTT for proteins containing the W3C mutation. The purity of the expressed proteins was analyzed by SDS-PAGE (15% gel) with 1x running buffer (25 mM Tris, 192 mM glycine, 0.5% SDS pH 8.3). PageRuler™ Prestained protein ladder, 10–180 kDa, (Thermo Scientific) was used as protein standard.

### Stability measurements using CD spectroscopy

CD spectra were recorded using a JASCO J-815 CD spectrometer with a JASCO PTC-423S/15 Peltier type thermostated cell holder. The thermal denaturation of parent scMN and mutants was monitored by recording the CD signal at 215 nm, in a 2 mm quartz cuvette, from 4 °C to 96 °C (scan rate 1 °C min^−1^, signal recorded every 1 °C, response time 16 s). The protein concentrations were 6 µM in 5 mM sodium phosphate buffer, pH 5.5, with 1 mM DTT added to samples containing proteins with the W3C mutations. Reversibility was monitored by recording a thermal scan from 96 °C to 4 °C directly after the forward scan. The secondary structures of the proteins were evaluated by far-UV CD spectroscopy before and after each thermal scan. Far-UV CD spectra were recorded between 190 and 250 nm, 50 nm/min, response 8 s, band with 1 nm, accumulations 3. A baseline recorded for only buffer was subtracted from each spectrum. Buffers were filtered through 0.2 µm filter and samples were centrifuged for 10 min at 13 000 rpm before measurements to remove possible aggregates.

The stability of parent scMN and mutants were modeled assuming a two-state reversible unfolding process. The temperature at the midpoint of unfolding (T_m_) and the enthalpy of unfolding (ΔH°_Tm_) were obtained by fitting equation (1) to CD data,

1 $$\begin{array}{rcl}{{\varepsilon }}_{{obs}} & = & (({{k}}_{{N}}\,\ast \,{T}+{{b}}_{{N}})+({{k}}_{{D}}\,\ast \,T+{{b}}_{{D}})\,\ast \,{{e}}^{-({\rm{\Delta }}{{H}}_{{Tm}}^{^\circ }(1-\frac{{T}}{{{T}}_{{m}}})+{\rm{\Delta }}{{C}}_{{p}}^{^\circ }({T}-{{T}}_{{m}}-{T}\ast \mathrm{ln}(\frac{{T}}{{{T}}_{{m}}})))/{RT}})\\  &  & /(1+{{e}}^{-({\rm{\Delta }}{{H}}_{{Tm}}^{^\circ }(1-\frac{{T}}{{{T}}_{{m}}})+{\rm{\Delta }}{{C}}_{{p}}^{^\circ }({T}-{{T}}_{{m}}-{T}\ast \mathrm{ln}(\frac{{T}}{{{T}}_{{m}}})))/{RT}})\,\end{array}$$

where ɛ_obs_ is the observed ellipticity at 215 nm, k_N_, b_N_, k_D_ and b_D_ define the baselines before and after the transition, T is the temperature in Kelvin, ΔH°_Tm_ is the enthalpy of unfolding, R is the gas constant and ΔC°_p_ is the difference in heat capacity between the denatured and native states. ΔC_p_ was assumed to be constant, 5858 J mol^−1^, which is reasonable for a protein of this size^53^.

Since the final plateaus were not reached for mutants S76Y, R39G + S76Y and W3C + R39G during temperature denaturation studies, the final plateau, X_FP_, was estimated by equation (2).

2 $${{X}}_{{FP}}={{Y}}_{{IP}}\frac{{parent}\,{M}{{N}}_{{FP}}}{{parent}\,{M}{{N}}_{{IP}}}$$

where Y_IP_ is the initial plateau for S76Y, R39G + S76Y or W3C + R39G before denaturation, parent MN_FP_ is the final plateau for parent scMN and MN_IP_ is the initial plateau for parent scMN.

The standard free energy of unfolding, ΔG°, was obtained by extrapolation from T_m_ to 25 °C by equation (3).

3 $${\rm{\Delta }}{G}^\circ ({T})={\rm{\Delta }}{H}^\circ ({{T}}_{{m}})[1-\frac{{T}}{{{T}}_{{m}}}]+{\rm{\Delta }}{{C}^\circ }_{{p}}[{T}-{{T}}_{{m}}-{T}\ast \mathrm{ln}(\frac{{T}}{{{T}}_{{m}}})]$$

### Sweet taste assessment

The sweetness of monellin mutants was assessed by six volunteers, three males and three females, ranging from 29 to 63 years of age with ethical approval via Lund University. The study was conducted in accordance with the ethics regulations of Lund University and informed consent was obtained from all participants. The study was one-way blinded (for the test persons) and the person preparing the samples was not present during the taste assay. The samples included parent scMN, R39G, S76Y, R39G + S76Y, MilliQ water (negative control) and sucrose (positive control). Samples were dissolved in MilliQ water. Mutants containing W3C were excluded due to requirement of 1 mM DTT in the samples. Dilution series of samples with concentrations 19, 29, 43, 65, 97, 146, 219, 328, 492, 738, 1108, 2492 and 5607 nM were tested starting from the lowest concentration. 1 mL of each sample was taken into the mouth and kept in the mouth for approximately 10 s then spit out. Before each new sample set testers rinsed their mouth with tap water until no sweet taste remained. A sample was recorded as sweet when the test person judged two consecutive samples as sweet. The detection threshold is reported as the lowest concentration where sweetness was detected.

All datasets generated and analysed during the current study are available from the corresponding author on reasonable request.

## Electronic supplementary material

Supplementary Information
