# Supplementary material for: Protein stabilization with retained function of monellin using a split GFP system
Source: Sci Rep. 2018 Aug 24;8:12763. doi: 10.1038/s41598-018-31177-z (PMC6109104; doi:10.1038/s41598-018-31177-z)
Supplement: Supplementary file 1 — Supplementary Information [file 41598_2018_31177_MOESM1_ESM.docx]

**Supplementary Information for**

**Protein stabilization with retained function of monellin using a split-GFP system**

Tanja Weiffert^a^, Sara Linse^a^

^a^Department of Biochemistry and Structural Biology, Lund University, Lund, 22100, Sweden.

e-mail: tanja.weiffert@biochemistry.lu.se, sara.linse@biochemistry.lu.se

**SI Materials and methods**

**Construction of a tandem NGFP-MNA-CGFP-MNB pQLinkN plasmid**

The original pQLinkN plasmid was a kind gift from Konrad Buessow (Addgene plasmid # 13670)^1^. Two pQLinkN plasmids containing the genes for NGFP-MNA and MNB-CGFP, respectively were purchased from Genscript. The NGFP-MNA and MNB-CGFP genes were then transferred from these two pQLinkN plasmids into the same plasmid (called NGFP-MNA-CGFP-MNB pQLinkN) using ligation independent cloning as previously described^1,2^. Shortly, the NGFP-MNA pQLinkN plasmid was digested with SwaI resctriction enzyme (New England Biolabs (NEB)) and the MNB-CGFP pQLinkN plasmid was digested with PacI (NEB). The restriction enzymes were heat inactivated and the digestion products were treated with T4 DNA polymerase (NEB) in the presence of either dGTP or dCTP (NEB). To anneal the plasmids, the digestion products were mixed and incubated at room temperature for 10 minutes before addition of ethylenediaminetetraacetic acid (EDTA) to a final concentration of 2.3 mM. Finally, the annealing product was incubated at 75°C for 1 min and cooled slowly to room temperature and transformed into Ca^2+^ competent *Escherichia coli* (*E. coli*) ER2566. Colonies growing on LB/agar containing 100 µg/mL ampicillin were picked, amplified and purified using GeneJET Plasmid Miniprep kit (Life Technologies). To identify plasmids containing the insert for both NGFP-MNA and MNB-CGFP, PCR with primers complementary to the sequences flanking MNA and MNB were performed. The PCR was performed using Expand High Fidelity PCR system (Roche) and the result was analyzed on an agarose gel to check for bands of the appropriate length. The following primers were used: epNGFPpQLinkNfor: 5’- GC TCT GGC TCG AGC GAG CTC -3’, epNGFPpQLinkNrev: 5’- C CAA GCT CAG CTA ATT AAG CTT GGT ACC TTA– 3’, epCGFPpQLinkNfor: 5’-GAG GAG AAA TTA ACT ATG GGA TCC CAT ATG-3’ and epCGFPpQLinkNrev: 5’-ACC CGA CGT CCC CTG CAG-3’. A NGFP-MNA-CGFP-MNB pQLinkN plasmid, containing both NGFP-MNA and MNB-CGFP, was selected and verified by sequencing (purchased of BM Unit).

**Mass spectrometry**

Protein samples were digested with trypsin (1:50 w/w, trypsin:protein) at 37°C overnight. Before the mass spectrometry analysis the peptides were cleaned up on reversed phase micro columns. The peptide samples were separated on reversed phase nano liquid chromatography (nano-LC) coupled to an LTQ-Orbitrap Velos Pro mass spectrometer (Thermo Fisher Scientific, Stockholm, Sweden) equipped with a nanoEasy spray ion source (Proxeon Biosystems, Odense, Denmark). The chromatographic separation was performed at 40ºC on a 15 cm (75 μm i.d.) EASY-Spray column packed with 3 μm resin. The nano high performance liquid chromatography (nano-HPLC) intelligent flow control gradient was created by solvent A (0.1% (v/v) FA in water) and solvent B (0.1% (v/v) FA in 100% (v/v) acetonitrile) and the gradient was run as follow; 5−30% solvent B over 40 min, from 30−50% solvent B over 20 min and from 50−95% solvent B over 5 min and at 95% solvent B for 10 min with a flow-rate of 300 nl/min. A full MS scan (mass range 400–1400 m/z, resolution of 60,000) was followed by MS/MS scans of the top 4 ions (resolution 7500) recorded in the orbitrap mass analyzer. The precursor ions were fragmented with collision induced dissociation (CID) at a normalized collision energy of 35 eV; activation time of 10 ms, isolation width, 3.0 m/z; ion transfer tube temperature, 275 °C; repeat count, 2; repeat duration, 30 s; exclusion duration, 600 s and automatic gain control (AGC) was set to 1e6 ions for both MS and MS/MS.

Raw data files were converted to mgf-format by Mascot Distiller (version 2.6) and identification of proteins were carried out with the Mascot Daemon software (version 2.4). The following search settings were used: trypsin as protease, 1 allowed missed cleavage sites, 10 ppm MS accuracy for peptides and 0.015 Da MS/MS accuracy, variable modifications: Oxidation (M). The files were searched against an in house created database containing the parent monellin as well as the different mutated monellin amino acid sequences. To be considered a true hit the individual peptide score > 25 and ions containing the mutation had to be detected.

**FarUV CD spectroscopy**

CD spectra were recorded from 250 to 190 nm using a JASCO J-815 CD spectrometer (Jasco Corporation) with a JASCO PTC-423S/15 Peltier type thermostated cell holder. CD spectra were recorded at 20°C in a 2 mm cuvette with an average of 3 scans, scan rate was 20 nm/min, response 8 s, bandwidth 1 nm and resolution 1 nm. Protein concentrations were 6 µM in 5 mM sodium phosphate buffer, pH 5.5.

**NMR spectroscopy**

1D ^1^H NMR spectra of 16 µM parent scMN and S76Y in water pH 6.25, with 10% D_2_O, were acquired using 1024-4096 scans on a 500 MHz Agilent VNMRS DirectDrive spectrometer. ^1^H frequency was 499.86 MHz and the water were suppressed by gradient echo^3,4^. Measurements were performed in NMR tube single use 5x178 mm with separate caps without hole (code Z107373, Bruker).

**SI References**

1 Scheich, C., Kummel, D., Soumailakakis, D., Heinemann, U. & Bussow, K. Vectors for co-expression of an unrestricted number of proteins. *Nucleic Acids Res.* **35**, e43, (2007).

2 Alexandrov, A. *et al.* A facile method for high-throughput co-expression of protein pairs. *Mol. Cell. Proteomics* **3**, 934-938, (2004).

3 Hwang, T. L. & Shaka, A. J. Multiple-pulse mixing sequences that selectively enhance chemical exchange or cross-relaxation peaks in high-resolution NMR spectra. *J. Magn. Reson.* **135**, 280-287, (1998).

4 Dalvit, C. & Hommel, U. New pulsed field gradient NMR experiments for the detection of bound water in proteins. *J. Biomol. NMR* **5**, 306-310, (1995).

CD (mdeg)


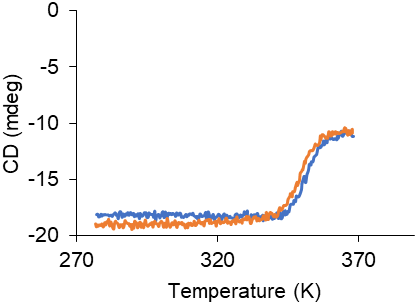

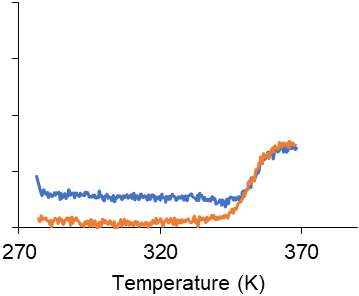

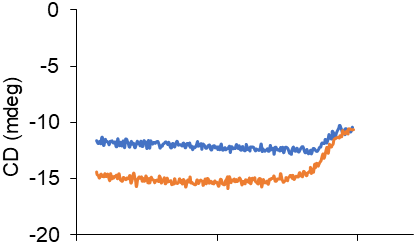

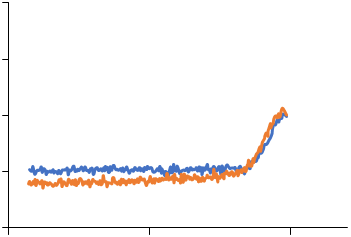

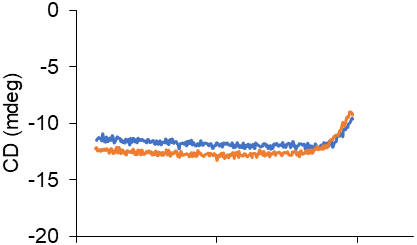

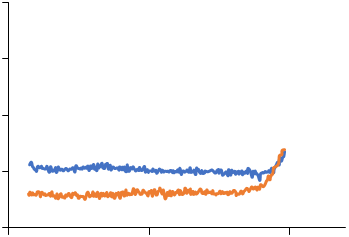

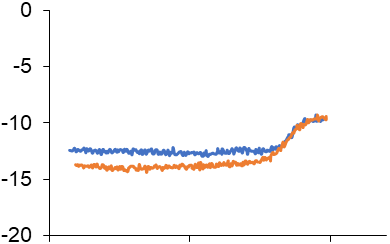

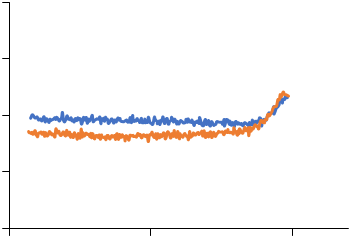


H

G

E

F

D

C

B

A

**Figure S1.** Reversibility of temperature denaturation followed by CD spectroscopy. Forward (blue) and reverse (orange) temperature denaturation of parent scMN (A), R39G + S76Y (B), S76Y (C), W3C + R39G (D), R39G (E), W3C + S76Y (F), W3C (G), W3C + R39G + S76Y (H) followed by CD spectroscopy.

**Table S1.** Sweet taste assessment results.

| Person | parent scMN (nM) | | S76Y (nM) | R39G (nM) | R39G + S76Y (nM) |
| --- | --- | --- | --- | --- | --- |
| 1 | | 219 | 219 | 5607 | * |
| 2 | | 328 | 219 | 1108 | 1108 |
| 3 | | 328 | 219 | 2492 | 2492 |
| 4 | | 219 | 328 | 5607 | 5607 |
| 5 | | 492 | 328 | 5607 | * |
| Mean | | 317 ± 112 | 262 ± 60 | 4084 ± 2141 | 3069 ± 2304 |
| ** indicates that no sweetness was detected.* | | | | | |
